# Supplementary material for: Identification of a novel mechanism of blood–brain communication during peripheral inflammation via choroid plexus‐derived extracellular vesicles
Source: EMBO Mol Med. 2016 Sep 5;8(10):1162–83. doi: 10.15252/emmm.201606271 (PMC5048366; doi:10.15252/emmm.201606271)
Supplement: Supplementary file 1 — Appendix [file EMMM-8-1162-s001.pdf]

Table of contents

Appendix Figure S1
Appendix Figure S2
Appendix Figure S3
Appendix Table S1
Appendix Table S2

Appendix Figure S1

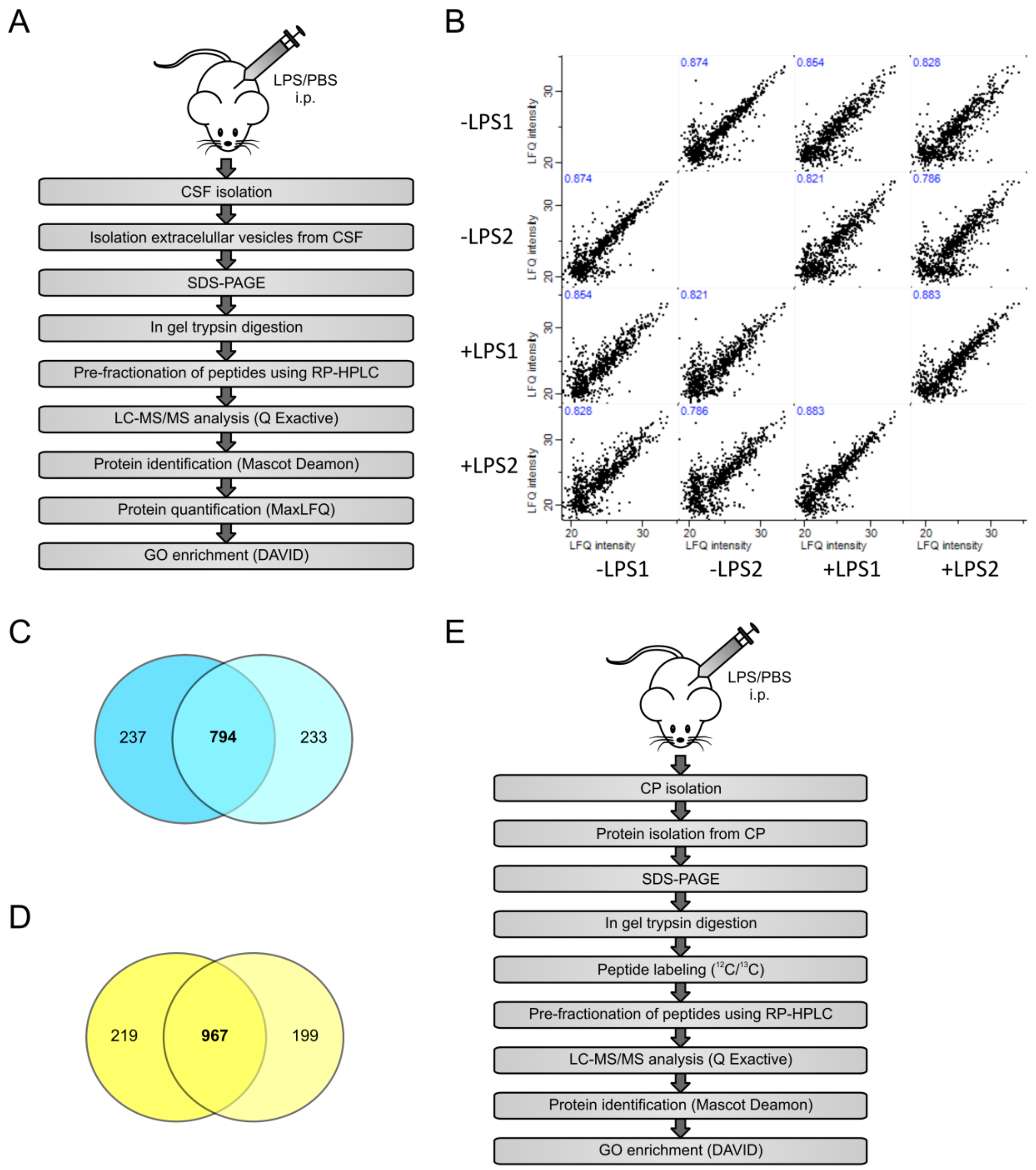

Fig. S1. Proteome analysis of the CPE-derived EVs and the choroid plexus in the presence and absence of LPS. (A) Schematic representation of the EV proteomics workflow. Mice were injected i.p. with PBS or LPS and 6 h later CSF was isolated using the cisterna magna puncture technique. Fifty microliters of CSF was pooled and EVs were isolated using the Total Exosome Isolation reagent (Life Technologies). Isolated EVs were separated by SDS-PAGE, stained with Coomassie brilliant blue, and digested with trypsin. After pre-fractionation using RP-HPLC, peptides were analyzed by LC-MS/MS (Q-Exactive; Thermo Fisher Scientific). Proteins were identified by using the Mascot Daemon software and quantified (label-free quantification) by MaxLFQ (MaxQuant). Finally, the data were analyzed using DAVID GO enrichment. **(B)** Scatter plots displaying LFQ intensities of the different

proteome samples. The calculated correlation coefficient (Pearson correlation) for each pair of samples is indicated in blue at the left corner of the graphs. '-LPS1' and '-LPS2' are biological replicates derived from EVs purified from CSF of PBS-injected mice. '+LPS1' and '+LPS2' are biological replicates derived from EVs purified from CSF of LPS-injected mice. **(C-D)** Overlap of proteins identified in EVs isolated from two independent pooled CSF samples (total volume 50 µl) of control (C; blue) and LPS-injected mice (D; yellow). **(E)** Schematic representation of the choroid plexus proteomics workflow. Mice were injected i.p. with PBS or LPS and 6 h later the choroid plexus (CP) was isolated. Choroid plexus tissue was lysed by mechanical disruption in Laemmli buffer followed by high speed centrifugation to remove the debris. Supernatant was collected, separated by SDS-PAGE, stained with Coomassie brilliant blue, and excised protein bands were digested with trypsin. Next, the peptide mixture was labeled with freshly prepared 12C (light; PBS) or 13C (heavy; LPS) N-hydroxy succinimide propionate, excess labeling was quenched, and light and heavy labeled samples were mixed in a 1:1 ratio. After pre-fractionation using RP-HPLC, the peptide samples were analyzed by LC-MS/MS (Q-Exactive; Thermo fisher scientific). Proteins were identified by using Mascot Daemon software, and the data were analyzed using DAVID.

Appendix Figure S2

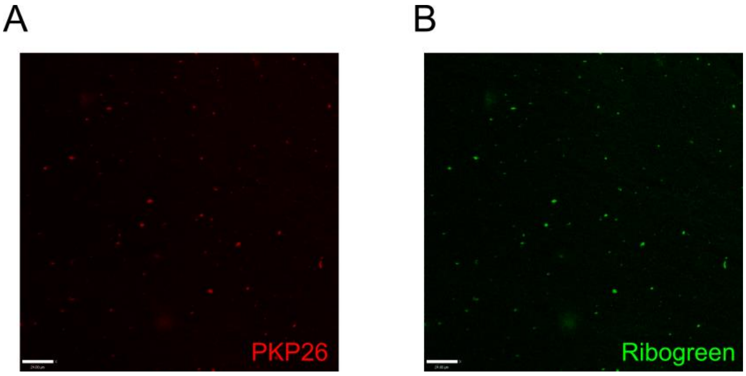

Fig. S2. Analysis of fluorescent labeling of the EVs. (A-B) Double-labelling of CSF-isolated EVs with the membrane label PKH26 (A; red) and RNA label Ribogreen (B; green).

Appendix Figure S3

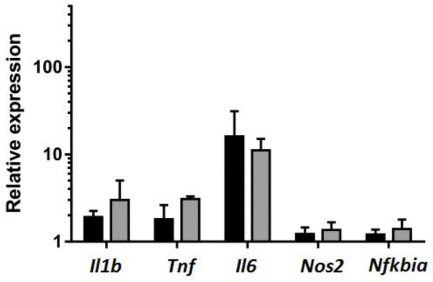

Fig. S3. Analysis of TLR4 dependency of the EV effect on recipient cells. QPCR gene expression analysis of inflammatory genes in wild type (black) and TLR4$^{-/-}$ (grey) derived primary mixed cortical cultures incubated for 24 h with EVs derived from LPS-injected mice (n=3).

Appendix Table S1

Primer	Sequence (5'-3')
***Anxa5*_Fw**	ATCCTGAACCTGTTGACATCCC
***Anxa5*_Rev**	AGTCGTGAGGGCTTCATCATA
***Calm2*_Fw**	ACGGGGATGGGACAATAACAA
***Calm2*_Rev**	TGCTGCACTAATATAGCCATTGC
***Cd63*_Forward**	GAAGCAGGCCATTACCCATGA
***Cd63*_Reverse**	TGACTTCACCTGGTCTCTAAACA
***Cd81*_Fw**	GTGGAGGGCTGCACCAAAT
***Cd81*_Rev**	GACGCAACCACAGAGCTACA
***Cd9*_Fw**	ATGCCGGTCAAAGGAGGTAG
***Cd9*_Rev**	GCCATAGTCCAATAGCAAGCA
***Dicer1*_Fw**	GGTCCTTTCTTTGGACTGCCA
***Dicer1*_Rev**	GCGATGAACGTCTTCCCTGA
***Dnmt3a*_Fw**	GAGGGAACTGAGACCCCAC
***Dnmt3a*_Rev**	CTGGAAGGTGAGTCTTGGCA
***Gapdh*_Fw**	TGAAGCAGGCATCTGAGGG
***Gapdh*_Rev**	CGAAGGTGGAAGAGTGGGAG
***Hprt*_Fw**	AGTGTTGGATACAGGCCAGAC
***Hprt*_Rev**	CGTGATTCAAATCCCTGAAGT
***Hspa1a*_Fw**	TCTCGGCACCACCTACTCC
***Hspa1a*_Rev**	CTACGCCCGATCAGACGTTT
***Nfkbia*_Fw**	CTCACGGAGGACGGAGACTC
***Nfkbia*_Rev**	CTCTTCGTGGATGATTGCCA
***Il1b*_Fw**	CACCTCACAAGCAGAGCACAAG
***Il1b*_Rev**	GCATTAGAAACAGTCCAGCCCATAC
***Il6*_Fw**	GCCTAAGCATATCAGTTTGTGGAC
***Il6*_Rev**	AGTGTCCCAACATTCATATTGTCAG
***Nos2*_Fw**	CAGCTGGGCTGTACAAACCTT
***Nos2*_Rev**	CATTGGAAGTGAAGCGTTTCG
***Irak1*_Fw**	AGCCGAGGTCTGCATTACATT
***Irak1*_Rev**	TGGCAGTCTGGATAACTGATGA
***Mapk3*_Fw**	TCCGCCATGAGAATGTTATAGGC
***Mapk3*_Rev**	GGTGGTGTTGATAAGCAGATTGG
***Notch1*_Fw**	CCCTTGCTCTGCCTAACGC
***Notch1*_Rev**	GGAGTCCTGGCATCGTTGG
***Smad2*_Fw**	ATGTCGTCCATCTTGCCATTC
***Smad2*_Rev**	AACCGTCCTGTTTTCTTTAGCTT
***Smad5*_Fw**	TTGTTCAGAGTAGGAACTGCAAC
***Smad5*_Rev**	GAAGCTGAGCAAACTCCTGAT
***Sox2*_Fw**	GCGGAGTGGAAACTTTTGTCC
***Sox2*_Rev**	CGGGAAGCGTGTACTTATCCTT
***Tab2*_Fw**	CATGACCTGCGACAAAAATTCC

***Tab2*_Rev**	TGATTGCGTAGACCAGAAATTCC
***Tnf*_Fw**	ACCCTGGTATGAGCCCATATAC
***Tnf*_Rev**	ACACCCATTCCCTTCACAGAG
***Ubc*_Fw**	AGGTCAAACAGGAAGACAGACGTA
***Ubc*_Rev**	TCACACCCAAGAACAAGCACA

Appendix Table S1. Forward (Fw) and reversed (Rev) primer sequences for qPCR.

Appendix Table S2

Figure	panel	comparison	p-value	n-value
**Figure 1**	B	2 h vs 0 h	0,03170	n = 5
		4 h vs 0 h	0,00790	n = 5
		6 h vs 0 h	0,00790	n = 5
	D		0,02860	n = 4
	F	1 h vs 0 h	0,02860	n = 4
		6 h vs 0 h	0,02860	n = 4
**Figure 2**	A		0,02680	n = 5
	C		0,03250	n = 3
	D		0,02100	n = 3 and n =4
	E		0,01640	n = 4
	G		0,00970	n = 3
	H		0,00030	n = 3
	I		0,00160	n = 3
**Figure 3**	B		0,02290	n = 3
	D		0,02250	n = 3
	E		0,03630	n = 3
	F		0,00950	n = 3
**Figure 4**	D	3 h vs 0 h	0,02830	n = 21 and n = 20
		4 h vs 0 h	< 0,0001	n = 13 and n = 20
		6 h vs 0 h	0,00960	n = 23 and n = 20
	E	3 h vs 0 h	< 0,0001	n = 21 and n = 20
		4 h vs 0 h	0,00260	n = 13 and n = 20
		6 h vs 0 h	< 0,0001	n = 23 and n = 20
	F	3 h vs 0 h	0,0002	n = 21 and n = 20
		4 h vs 0 h	< 0,0001	n = 13 and n = 20
		6 h vs 0 h	0,0004	n = 23 and n = 20
	G	1 h vs 0 h	0,00840	n = 5 and n = 4
		6 h vs 0 h	0,00450	n = 5 and n = 4
		24 h vs 0 h	0,03680	n = 3 and n = 4
	H	1 h vs 0 h	0,00170	n = 3
		24 h vs 0 h	0,00120	n = 3
	I	6 h vs 0 h	0,01780	n = 5 and n = 4
		24 h vs 0 h	0,01140	n = 3 and n = 4
	J	1 h vs 0 h	0,00830	n = 5 and n = 4
		6 h vs 0 h	0,00030	n = 5 and n = 4
		24 h vs 0 h	0,00050	n = 3 and n = 4
	K		0,04400	n = 8 and n = 10
	L	mR-9	0,04090	n = 4
		miR-146a	0,03719	n = 4
		miR-155	0,03032	n = 4
	M		0,00660	n = 7
**Figure 5**	C		0,00210	n = 3 and n = 4
	D	miR-9	0,04427	n = 6 and n = 5
		miR-155	0,00061	n = 5 and n = 7
	E	miR-9	0,02126	n = 5 and n = 4
		miR-155	0,03627	n = 5 and n = 4

	F		0,01710	n = 5 and n = 4
	G	miR-9	0,00820	n = 5 and n = 7
		miR-146a	0,00574	n = 6
		miR-155	0,00000	n = 7
	H	miR-146a	0,02993	n = 3
		miR-155	0,04198	n = 3
**Figure 8**	A	*Mapk3*	0,00280	n = 3
		Notch1	0,02234	n = 3
		Dicer1	0,03244	n = 3
		Tab2	0,00666	n = 3
		Sox2	0,00729	n = 3
		Smad2	0,01496	n = 3
		Smad5	0,00178	n = 3
		Irak1	0,03244	n = 3
	B	*Notch1*	0,03290	n = 3
		Dicer1	0,04111	n = 3
		Smad2	0,04737	n = 3
		Smad5	0,04486	n = 3
		Dnmt3a	0,00884	n = 3
	C *in vitro*	*Il1b*	0,00181	n = 3
		Tnf	0,00588	n = 3
		Il6	0,00395	n = 3
		Nos2	0,00258	n = 3
		Nfkbia	0,00215	n = 3
	C *in vivo*	*Il1b*	0,00149	n = 3
		Tnf	0,01980	n = 3
		Il6	0,00262	n = 3
		Nos2	0,02425	n = 3
		Nfkbia	0,00771	n = 3
	D *in vitro*	IL-6	0,01131	n = 3
		IL-1β	0,00009	n = 3
		TNF	0,00747	n = 3
	D *in vivo*	IL-6	0,01346	n = 3 and n= 5
		IL-1β	0,01190	n = 3 and n= 8
		TNF	0,00304	n = 3 and n= 8
	E	*Nfkbia*	0,00686	n = 3
	F	*Dnmt3a*	0,00505	n = 3
	G	*Dicer1*	0,04714	n = 6 and n = 7
		Tab2	0,03036	n = 6 and n = 8
		Sox2	0,00344	n = 6 and n = 7
		Dnmt3a	0,04355	n = 6 and n = 8
		Irak1	0,00053	n = 6 and n = 8
	H	*Il1b*	0,03524	n = 6 and n = 7
		Tnf	0,00294	n = 6 and n = 8
		Nos2	0,02709	n = 6
		Nfkbia	0,00035	n = 3
**Figure EV1**	E	2 h vs 0 h	0,00090	n = 6
		3 h vs 0 h	0,00060	n = 6
		4 h vs 0 h	0,00010	n = 6

		5 h vs 0 h	< 0,0001	n = 6
		6 h vs 0 h	< 0,0001	n = 6
		7 h vs 0 h	< 0,0001	n = 6
		8 h vs 0 h	< 0,0001	n = 6
		9 h vs 0 h	< 0,0001	n = 6
		10 h vs 0 h	< 0,0001	n = 6
**Figure EV4**	A		0,01180	n = 3 and n = 4
	C		0,00210	n = 3
	D		0,02280	n = 3
	E		0,02350	n = 3 and n = 4
**Figure EV5**	B		0,04230	n = 3 and n = 5

Appendix Table S2. Overview of the exact p- and n-values of the significant results.
