# Supplementary material for: Identification of a novel mechanism of blood–brain communication during peripheral inflammation via choroid plexus‐derived extracellular vesicles
Source: EMBO Mol Med. 2016 Sep 5;8(10):1162–83. doi: 10.15252/emmm.201606271 (PMC5048366; doi:10.15252/emmm.201606271)
Supplement: Supplementary file 2 — Expanded View Figures PDF [file EMMM-8-1162-s002.pdf]

Expanded View Figures

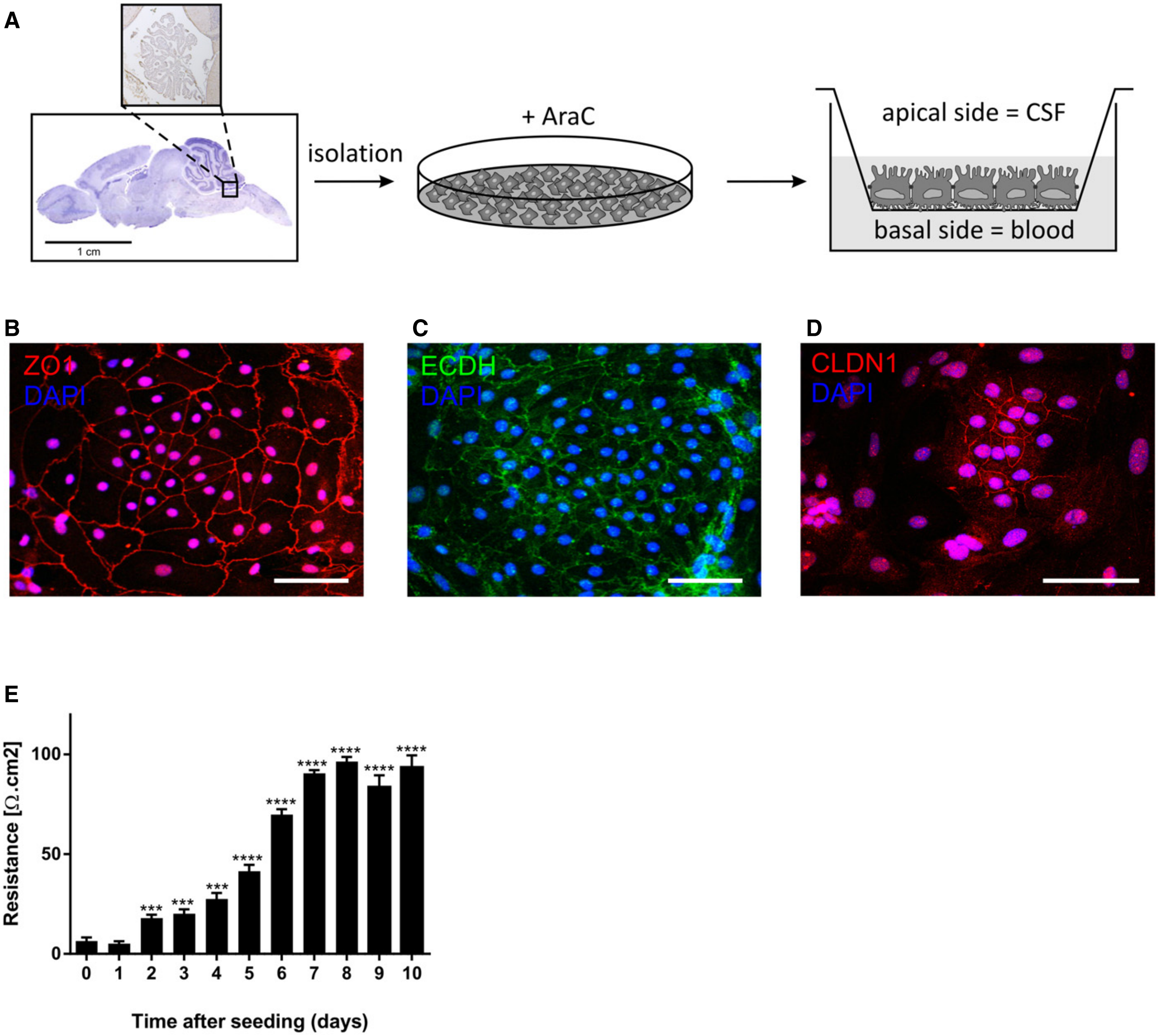

Figure EV1. Characterization of the primary CPE cell culture.

A Schematic representation of the procedures of primary CPE cell isolation and culture in the transwell system.

B, D Representative confocal images showing the expression of zona occludens (ZO1, red, B), E-cadherin (ECDH, green, C), and claudin-1 (CLDN1, red, D) in the primary CPE culture. Scale bars, 30 μm.

E TEER values of the primary CPE cells grown in the transwell system. Significance was calculated compared with day 0 (n = 6). Data are presented as means ± SEM. Data were analyzed by Student's t-test. Significance levels are indicated on the graphs: ***$0.0001 \leq P < 0.001$; ****$P < 0.0001$.

© 2016 The Authors

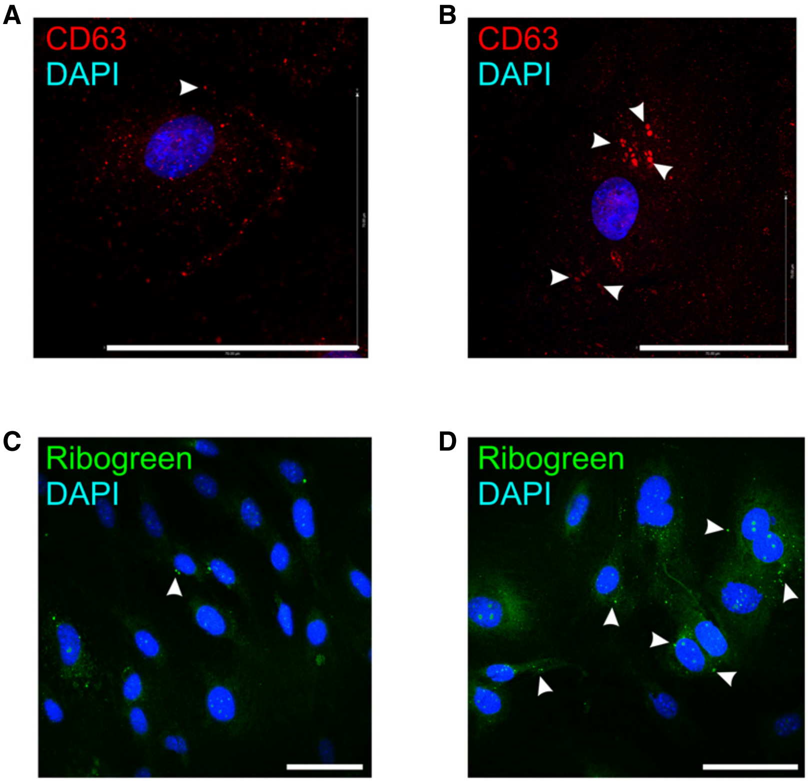

Figure EV2. Analysis of the exosomal machinery in the CPE cells upon systemic inflammation.

A–D Representative CD63 (red, A, B) and RiboGreen (green, C, D) staining of primary CPE cells after 12 h in the absence (A, C) or presence (B, D) of LPS *in vitro* from two independent experiments with $n = 3$. The white arrows indicate CD63 (A, B) or RiboGreen (C, D) positive vesicles. Scale bars, 70 μm.

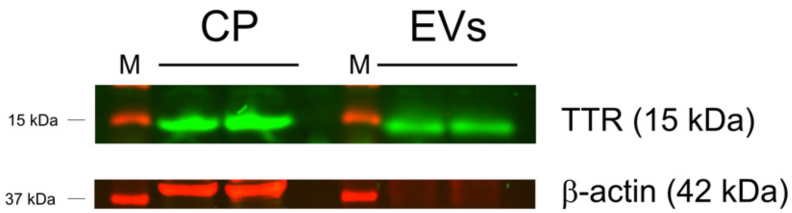

Figure EV3. Western blot analysis of choroid plexus cell lysates and EVs isolated from CSF. Choroid plexus (CP) tissue was isolated, pooled from three mice, lysed, and analyzed by SDS–PAGE. Similarly, EVs were isolated from ~25 μl CSF and analyzed by SDS–PAGE. Detection was done with an anti-TTR antibody (green) and an anti-β-actin antibody (red) using the Odyssey Imaging system.

Figure EV4. Analysis of the exosomal machinery in CPE cells upon systemic inflammation.

A–E QPCR gene expression analysis of exosomal markers *Cd9* (A), *Cd81* (B), *Hspa1a* (C), *Cd63* (D), and *Anxa5* (E) in the choroid plexus before and after LPS treatment ($n = 4$). Data are displayed as mean ± SEM and analyzed by Student's *t*-test. Significance levels are indicated on the graphs: $^{*}0.01 \le P < 0.05$; $^{**}0.001 \le P < 0.01$.

F–K Representative TEM images from choroid plexus tissue isolated 0 (F), 1 (G), 2 (H), 3 (I), 4 (J), or 6 (K) h after LPS injection. Black arrow heads point to exosomes present in MVBs. Scale bars, 9 μm. Mv, microvilli; Nu, nucleus.

L–N *In situ* hybridization (ISH) analysis of miR expression in CPE cells *in vivo*. LNA™-ISH of miR-146a (L), miR-9 (M), and miR-155 (N) on brain sections. Scale bars, 30 μm.

© 2016 The Authors

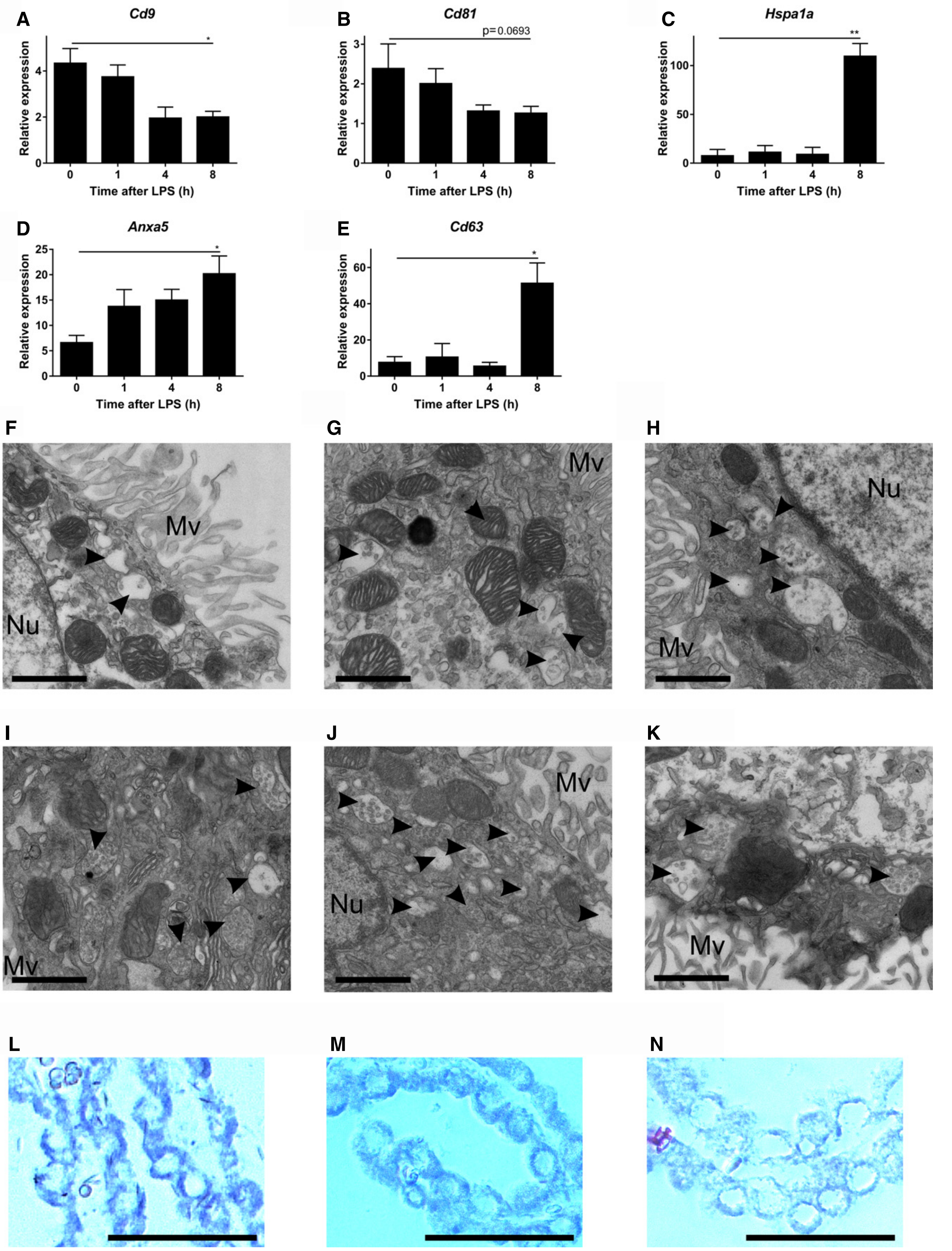
A
Cd9
*
Relative expression
0
2
4
0
1
4
8
Time after LPS (h)
B
Cd81
p=0.0693
Relative expression
0
1
2
3
0
1
4
8
Time after LPS (h)
C
Hspa1a
**
Relative expression
0
50
100
0
1
4
8
Time after LPS (h)
D
Anxa5
*
Relative expression
0
5
10
15
20
25
0
1
4
8
Time after LPS (h)
E
Cd63
*
Relative expression
0
20
40
60
0
1
4
8
Time after LPS (h)
F
Mv
Nu
G
Mv
H
Nu
Mv
I
Mv
J
Mv
Nu
K
Mv
L
M
N

Figure EV4.

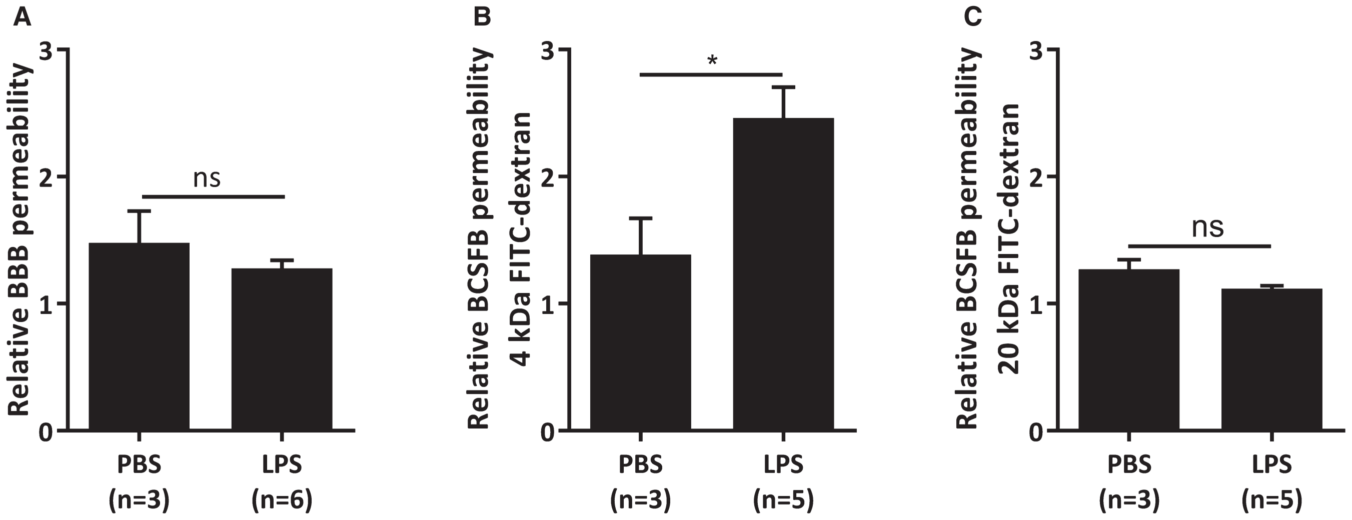

Figure EV5. Analysis of blood–brain barrier (BBB) and blood-CSF barrier (BCSFB) leakage upon systemic inflammation.

A–C Relative BBB (A) and BCSFB (B, C) leakage upon LPS injection. Mice were injected with PBS or LPS, followed 3 h later by i.v. injection of 4 kDa (A, B) or 20 kDa (C) FITC-dextran. One hour later, CSF was isolated and fluorescence was measured using a fluorometer to determine the relative BCSFB leakage ($\lambda_{ex}/\lambda_{em}$ = 488 nm/520 nm). Additionally, brains were isolated and incubated in formamide overnight at 37°C. The next day, the supernatant was isolated and fluorescence was measured using a fluorometer ($\lambda_{ex}/\lambda_{em}$ = 488 nm/520 nm) to determine the relative BBB leakage. Data are displayed as mean ± SEM and analyzed by Student's *t*-test. Significance levels are indicated on the graphs: $*0.01 \leq P < 0.05$.

© 2016 The Authors
